# Recent Developments in the Viscosity Modeling of Concentrated Monodisperse Emulsions

**DOI:** 10.3390/foods12183483

**Published:** 2023-09-19

**Authors:** Rajinder Pal

**Affiliations:** Department of Chemical Engineering, University of Waterloo, Waterloo, ON N2L 3G1, Canada; rpal@uwaterloo.ca; Tel.: +1-519-888-4567 (ext. 32985)

**Keywords:** rheology, viscosity, relative viscosity, viscosity models, capillary number, emulsion, dispersion, droplets, oil-in-water, water-in-oil

## Abstract

Emulsions form a large group of food materials. Many foods are either partly or wholly emulsions or are in the form of emulsion at some stage of the production process. A good understanding of the rheological properties of emulsions, especially their shear viscosity, is essential in the design, formulation, and processing of food emulsions. The texture and mouthfeel of food emulsions are also largely influenced by emulsion viscosity. Therefore, it is of practical importance to be able to correlate and predict emulsion viscosity as a function of droplet concentration and other relevant variables. In this article, the recent developments made in the viscosity modeling of concentrated emulsions are reviewed. The viscosity models for concentrated emulsions published in the twenty-first century are discussed, compared, and evaluated using a large body of experimental viscosity data available on emulsions. The effects of droplet size distribution and capillary number on the viscosity of concentrated emulsions are also discussed in detail. A new generalized viscosity model is developed for concentrated emulsions that includes the effect of capillary number and is accurate with small average percent relative error (within 3%).

## 1. Introduction

Emulsions are heterogeneous mixtures of immiscible oil and aqueous phases. Oil-in-water (abbreviated O/W) emulsions consist of oil droplets distributed in a continuum of aqueous phase. Water-in-oil (abbreviated W/O) emulsions consist of water droplets distributed in a continuum of oil phase [1]. Emulsions are very important in food applications [1,2,3,4,5,6,7,8]. Many delicious foods are either partly or wholly emulsions or are in emulsion form at some stage of the production process. For example, mayonnaise is an O/W emulsion consisting of vegetable oil droplets suspended in an aqueous phase containing egg yolks, lemon juice or vinegar, and mustard. The fat (oil droplets) content of mayonnaise ranges from about 70 to 80 percent. Other examples of food emulsions are butter, cream, cake batters, coffee whitener, desserts, fruit beverages, ice cream, margarine, milk, soups, sausages, sauces, and salad dressings. Many food emulsions are complex fluids consisting of additives such as sugar, salts, vitamins, minerals, food-grade surfactants, proteins, gums, colors, and flavors, in addition to oil and water. However, the most important components of food emulsions from a rheological point of view are their oil and water content. Table 1 gives examples of the composition of some emulsion-based food products.

An understanding of the rheology of emulsions is essential in the design and formulation of emulsion-type foods with desired sensory, nutritional, and physicochemical properties [2]. The design and operation of equipment required to mix, process, transport, store, and pump emulsion-based foods also require a thorough understanding of the rheology of emulsions.

The rheology of food emulsions can be complex depending upon the composition of the food. In general, food emulsions exhibit the following rheological properties: shear viscosity, elastic modulus, and yield stress. However, the most important rheological property of food emulsions is their shear viscosity. The shear viscosity (simply viscosity) of food emulsions is a function of factors such as the viscosity of the matrix phase, the ratio of droplet viscosity to matrix viscosity, the concentration of the dispersed phase (droplets), droplet size distribution, and shear rate. Food manufacturers are interested in the measurement, prediction, control, and manipulation of shear viscosity of food emulsions as viscosity affects the appearance, texture, mouthfeel, and processability of emulsion-based foods.

The focus of this article is the shear viscosity of emulsions. The recent developments in the modeling of the viscosity of concentrated emulsions are discussed. The viscosity models for concentrated emulsions published in the twenty-first century are reviewed, compared, and evaluated using a large body of experimental viscosity data available on concentrated emulsions.

## 2. Theoretical Background

### 2.1. Infinitely Dilute Emulsions of Identical Droplets

For an infinitely dilute emulsion consisting of identical droplets (same size, shape, and material), the rheological equation of state can be written as [1]:

(1) $$\overset{̿}{\sigma }={\overset{̿}{\sigma }}_{o}+\left(\frac{3\varphi }{4\pi R^{3}}\right){\overset{̿}{S}}^{o}$$

where $\overset{̿}{\sigma }$ is the stress tensor in an emulsion, ${\overset{̿}{\sigma }}_{o}$ is the stress tensor in the suspending medium (matrix) at the same rate of strain tensor as that imposed on an emulsion, ${\overset{̿}{S}}^{o}$ is the dipole strength of a spherical droplet, φ is the volume fraction of droplets, and R is the radius of droplets.

The dipole strength of a spherical droplet is given as [1]:

(2) $${\overset{̿}{S}}^{\;o}=\frac{4}{3}\pi R^{3}\eta_{m}\left(\frac{2+5\lambda }{1+\lambda }\right)\overset{̿}{E}$$

where $\eta_{m}$ is the matrix fluid viscosity, λ is the ratio of droplet viscosity to matrix fluid viscosity, and $\overset{̿}{E}$ is the imposed rate of strain tensor. Note that this expression of dipole strength is valid at a low capillary number (Ca) where Ca is defined as:

(3) $$Ca=\frac{\eta_{m}R\dot{\gamma }}{\gamma }$$

γ is the interfacial tension between the two liquids (oil and water) and $\dot{\gamma }$ is the imposed shear rate. Equation (2) is valid in the limit Ca→0 where the droplet is spherical in shape.

Assuming the matrix fluid to be incompressible Newtonian, the stress tensor in the suspending medium or matrix fluid can be expressed as:

(4) $${\overset{̿}{\sigma }}_{o}=-P\overset{̿}{\delta }+2\eta_{m}\overset{̿}{E}$$

where P is pressure. From Equations (1), (2) and (4), it can be readily shown that:

(5) $$\overset{̿}{\sigma }=-P\overset{̿}{\delta }+2\eta_{m}\overset{̿}{E}\left[1+\left(\frac{2+5\lambda }{2+2\lambda }\right)\varphi \right]$$

This is the rheological constitutive equation for an infinitely dilute emulsion valid at low capillary numbers (Ca→0). From Equation (5), it follows that the viscosity of an infinitely dilute emulsion of spherical droplets is:

(6) $$\eta =\eta_{m}\left[1+\left(\frac{2+5\lambda }{2+2\lambda }\right)\varphi \right]$$

where η is emulsion viscosity. Equation (6) is the celebrated Taylor equation [9] for the viscosity of an infinitely dilute emulsion of spherical droplets. The Taylor equation was published in 1934.

Frankel and Acrivos [10] extended the Taylor relationship, Equation (6), to non-zero capillary numbers by considering the first-order deformation of droplets in the shear field. They derived the following expression for the relative viscosity of infinitely dilute emulsions:

(7) $$\eta_{r}=\frac{\eta }{\eta_{m}}=\frac{1}{1+{\left(hCa\right)}^{2}}\left[1+\left(\frac{2+5\lambda }{2+2\lambda }\right)\varphi +{\left(hCa\right)}^{2}\left\{1+\left(\frac{2+5\lambda }{2+2\lambda }\right)\varphi -\frac{19\lambda +16}{\left(2\lambda +2\right)\left(2\lambda +3\right)}\varphi \right\}\right]$$

where $\eta_{r}$ is the relative viscosity defined as the ratio of emulsion viscosity η to matrix fluid viscosity $\eta_{m}$ and h is given as follows:

(8) $$h=\frac{\left(19\lambda +16\right)\left(2\lambda +3\right)}{40\left(\lambda +1\right)}$$

Note that in the limit Ca→0, the Frankel and Acrivos equation, Equation (7), reduces to the Taylor equation, Equation (6).

According to the Frankel and Acrivos model, emulsions exhibit a shear-thinning behavior in that the emulsion viscosity decreases with the increase in shear rate or capillary number. The shear-thinning in emulsions is due to the orientation and elongation of droplets in the direction of flow with the increase in Ca.

### 2.2. Non-Dilute Emulsions of Identical Droplets

The Taylor model (Equation (6)) and the Frankel and Acrivos model (Equation (7)) were developed on the basis of a single-droplet mechanics. Hence, they are applicable to infinitely dilute emulsions. They do not take into consideration any interactions between the neighboring droplets. At a finite concentration of droplets, the hydrodynamic interactions between the droplets become important. Consequently, the viscosity of non-dilute emulsions is much higher than the values predicted by the Taylor or Frankel and Acrivos models.

Oldroyd [11] developed a model for the zero-shear (Ca→0) viscosity of non-dilute emulsions using an effective medium approach. The Oldroyd model is given as:

(9) $$\eta_{r}=\frac{1+\frac{3}{2}\left[\frac{5\lambda +2}{5\lambda +5}\right]\varphi }{1-\varphi \left[\frac{5\lambda +2}{5\lambda +5}\right]}$$

Upon expansion, the Oldroyd model can be re-written as:

(10) $$\eta_{r}=1+\left(\frac{2+5\lambda }{2+2\lambda }\right)\varphi +\frac{{\left(2+5\lambda \right)}^{2}}{10{\left(1+\lambda \right)}^{2}}\varphi^{2}+\ldots$$

In the limit φ→0, the Oldroyd model reduces to the Taylor equation. The Oldroyd model is an improvement over the Taylor model. However, it underpredicts the relative viscosities of emulsions when φ>0.10.

Yaron and Gal-Or [12] and Choi and Schowalter [13] utilized the cell model approach to develop the viscosity equations for non-dilute emulsions. In the limit of low capillary numbers (Ca→0), their equations are given as follows:

Yaron and Gal-Or:

(11) $$\eta_{r}=1+\varphi \left[\frac{5.5\left\{4\varphi^{7/3}+10-\left(84/11\right)\varphi^{2/3}+\left(4/\lambda \right)\left(1-\varphi^{7/3}\right)\right\}}{10\left(1-\varphi^{10/3}\right)-25\varphi \left(1-\varphi^{4/3}\right)+\left(10/\lambda \right)\left(1-\varphi \right)\left(1-\varphi^{7/3}\right)}\right]$$

Choi and Schowalter:

(12) $$\eta_{r}=1+\varphi \left[\frac{2\left\{\left(5\lambda +2\right)-5\left(\lambda -1\right)\varphi^{7/3}\right\}}{4\left(\lambda +1\right)-5\left(5\lambda +2\right)\varphi +42\lambda \varphi^{5/3}-5\left(5\lambda -2\right)\varphi^{7/3}+4\left(\lambda -1\right)\varphi^{10/3}}\right]$$

In the limit φ→0, the Yaron and Gal-Or model reduces to:

(13) $$\eta_{r}=1+2.2\left(\frac{2+5\lambda }{2+2\lambda }\right)\varphi$$

Thus, the Yaron and Gal-Or model does not reduce to the Taylor equation in the limit φ→0. However, the Choi and Schowalter model reduces to the Taylor equation in the limit φ→0.

The major drawback of the emulsion viscosity equations derived based on the cell model approach is that the equations derived are dependent on the size, shape, and boundary conditions of the chosen cell. The boundary conditions used in the derivations of Yaron and Gal-Or model and Choi and Schowalter model are different. Consequently, they give different predictions of the relative viscosity of emulsions.

## 3. Recent Developments in the Viscosity Modeling of Concentrated Emulsions

In this section, the viscosity models for concentrated monodisperse emulsions developed in the twenty-first century are reviewed, compared, and evaluated using a large body of experimental data available on the viscosity of concentrated monomodal emulsions.

Pal [14] used the effective medium theory to develop the following differential equation for concentrated monodisperse emulsions:

(14) $$\frac{d\eta }{d\varphi }=\frac{K_{o}\eta }{1-K_{o}\varphi }\left[\frac{\eta +2.5\eta_{d}}{\eta +\eta_{d}}\right]$$

where $\eta_{d}$ is the droplet viscosity and $K_{o}=1/\varphi_{m}$ ($\varphi_{m}$ is the maximum packing volume fraction of droplets). Upon the integration of Equation (14) with the boundary condition $\eta =\eta_{m}$ at φ=0, the following equation for the viscosity of concentrated monodisperse emulsions is obtained:

(15) $$\eta_{r}{\left[\frac{2\eta_{r}+5\lambda }{2+5\lambda }\right]}^{3/2}={\left(1-\frac{\phi }{\phi_{m}}\right)}^{-2.5}$$

This equation is referred to as model P1 in the remainder of this article.

Starting from the Taylor emulsion viscosity equation, Equation (6), and utilizing the effective medium approach along with the “crowding effect” of droplets, Pal [15] derived the following differential for concentrated monodisperse emulsions:

(16) $$d\eta =2.5\eta \left[\frac{0.4\eta +\eta_{d}}{\eta +\eta_{d}}\right]d\left(\frac{\varphi }{1-\frac{\phi }{\phi_{m}}}\right)$$

Upon the integration of this equation with the boundary condition $\eta =\eta_{m}$ at φ=0, the following equation for the viscosity of concentrated monodisperse emulsions is obtained:

(17) $$\eta_{r}{\left[\frac{2\eta_{r}+5\lambda }{2+5\lambda }\right]}^{3/2}=exp\left(\frac{2.5}{1-\frac{\phi }{\phi_{m}}}\right)$$

This equation is referred to as model P2 in the remainder of this article.

Pal [15] further contended that the differential equation, Equation (16), overcorrects for the crowding effect of droplets and, therefore, modified the differential equation, Equation (16), as follows:

(18) $$d\eta =2.5\eta \left[\frac{0.4\eta +\eta_{d}}{\eta +\eta_{d}}\right]\frac{d\varphi }{\left(1-\frac{\phi }{\phi_{m}}\right)}$$

Upon integration and using the boundary condition $\eta =\eta_{m}$ at φ=0, this equation gives:

(19) $$\eta_{r}{\left[\frac{2\eta_{r}+5\lambda }{2+5\lambda }\right]}^{3/2}={\left(1-\frac{\phi }{\phi_{m}}\right)}^{-2.5\varphi_{m}}$$

This equation is referred to as model P3 in the remainder of this article.

Pal [16] observed that the relative viscosity equations for suspensions of solid particles can be transformed into the relative viscosity equations for emulsions of liquid droplets by replacing $\eta_{r}$ with $\eta_{r}{\left[\left(2\eta_{r}+5\lambda \right)/\left(2+5\lambda \right)\right]}^{3/2}$. For example, the relative viscosity equation for a suspension of solid particles is given as:

(20) $$\eta_{r}=H\left(\varphi ,\varphi_{m}\right)$$

where $H\left(\varphi ,\varphi_{m}\right)$ is a relative viscosity function of suspension. This suspension equation can be transformed into emulsion viscosity equation as follows:

(21) $$\eta_{r}{\left[\frac{2\eta_{r}+5\lambda }{2+5\lambda }\right]}^{3/2}=H\left(\varphi ,\varphi_{m}\right)$$

Using this reasoning as the basis, Pal [16] proposed the following series of emulsion viscosity equations:

(22) $$\eta_{r}{\left[\frac{2\eta_{r}+5\lambda }{2+5\lambda }\right]}^{3/2}={\left(1+\frac{1.25\varphi }{1-\frac{\varphi }{\varphi_{m}}}\right)}^{2}$$

(23) $$\eta_{r}{\left[\frac{2\eta_{r}+5\lambda }{2+5\lambda }\right]}^{3/2}={\left(1-\frac{\varphi }{\varphi_{m}}\right)}^{-2}$$

(24) $$\eta_{r}{\left[\frac{2\eta_{r}+5\lambda }{2+5\lambda }\right]}^{3/2}={\left(1+\frac{0.75\left(\varphi /\varphi_{m}\right)}{1-\frac{\varphi }{\varphi_{m}}}\right)}^{2}$$

(25) $$\eta_{r}{\left[\frac{2\eta_{r}+5\lambda }{2+5\lambda }\right]}^{3/2}=\frac{9}{8}\left[\frac{{\left(\varphi /\varphi_{m}\right)}^{1/3}}{1-{\left(\varphi /\varphi_{m}\right)}^{1/3}}\right]\left(\varphi \geq 0.1042\varphi_{m}\right)$$

Equation (22) is referred to as model P4, Equation (23) as model P5, Equation (24) as model P6, and Equation (25) as model P7 in the remainder of this article.

Mendoza and Santamaria-Holek [17] utilized Pal’s approach to generalize their suspension viscosity model to emulsions as given below:

(26) $$\eta_{r}{\left[\frac{2\eta_{r}+5\lambda }{2+5\lambda }\right]}^{3/2}={\left(1-\frac{\varphi }{1-c\varphi }\right)}^{-2.5}\;where\;c=\frac{1-\varphi_{m}}{\varphi_{m}}$$

Equation (26) is referred to as model MS in the remainder of this article.

Faroughi and Huber [18] modified the Brouwers viscosity model for suspensions [19] to make it an emulsion viscosity model by replacing the Einstein coefficient of 2.5 in the exponent of the model by N as defined below:

(27) $${\eta_{r}=\left[\frac{\varphi_{m}-\varphi }{\varphi_{m}(1-\varphi )}\right]}^{-N\varphi_{m}/\left(1-\varphi_{m}\right)}\;where\;N=\left(\frac{2+5\lambda }{2+2\lambda }\right)$$

This equation is referred to as model FH in the remainder of this article.

The emulsion viscosity models discussed thus far are not explicit in relative viscosity $\eta_{r}$. Pal [20,21] modified the Einstein equation and developed the following models explicit in $\eta_{r}$ for concentrated monodisperse emulsions in the limit of Ca→0:

(28) $$\eta_{r}=1+\frac{5}{2}\left[\frac{\varphi_{eff}\left(\frac{2+5\lambda }{5+5\lambda }\right)}{1-\varphi_{eff}\left(\frac{2+5\lambda }{5+5\lambda }\right)}\right]$$

where:

(29) $$\varphi_{eff}=\left[1+\left(\frac{1-\varphi_{m}}{\varphi_{m}}\right)\left(\sqrt{1-{\left(\frac{\varphi_{m}-\varphi }{\varphi_{m}}\right)}^{2}}\right)\right]\varphi$$

(30) $$\eta_{r}=1+\frac{5}{2}\left(\frac{\varphi_{eff}}{1-\varphi_{eff}}\right)$$

where:

(31) $$\varphi_{eff}=\left[1+\left(\frac{1-\varphi_{m}}{\varphi_{m}}\right)\left(\sqrt{1-{\left(\frac{\varphi_{m}-\varphi^{*}}{\varphi_{m}}\right)}^{2}}\right)\right]\varphi^{*}$$

(32) $$\varphi^{*}=\left(\frac{2+5\lambda }{5+5\lambda }\right)\varphi$$

The clustering of droplets at non-dilute concentrations was taken into consideration through the effective volume fraction $\varphi_{eff}.$ The expression for $\varphi_{eff}$ was derived considering the following characteristics of emulsions: (a) $\varphi_{eff}=\varphi$ when φ→0; (b) $\varphi_{eff}=1$ when $\varphi \to \varphi_{m}$; (c) $\partial \left(\varphi_{eff}/\varphi \right)/\partial \varphi \geq 0$; and (d) $\partial \left(\varphi_{eff}/\varphi \right)/\partial \varphi =0$ when $\varphi \to \varphi_{m}$. Equation (28) in conjunction with Equation (29) is referred to as P8 model in the remainder of this article. Equation (30) in conjunction with Equations (31) and (32) is referred to as P9 model in the remainder of this article.

Pal [22] recently developed another viscosity model for concentrated monodisperse emulsions using the effective medium approach and taking into consideration the clustering of droplets in a shear field:

(33) $$\eta_{r}{\left[\frac{2\eta_{r}+5\lambda }{2+5\lambda }\right]}^{3/2}={\left(1-\varphi_{eff}\right)}^{-2.5}$$

where:

(34) $$\varphi_{eff}=\left\{1+\left[\frac{1-\varphi_{m}}{\varphi_{m}^{2}}\right]\varphi \right\}\varphi$$

This equation is referred to as model P10 in the remainder of this article. Note that the effective concentration expression given in Equation (34) is much simpler than that given in Equations (29) or (31).

Using the reasoning that the relative viscosity function of suspension $H\left(\varphi ,\varphi_{m}\right)$ is applicable to emulsions, the following new models for the viscosity of concentrated emulsions are proposed for the first time:

(35) $$\eta_{r}{\left[\frac{2\eta_{r}+5\lambda }{2+5\lambda }\right]}^{3/2}=1+2.5\varphi +10.05\varphi^{2}+0.00273exp(16.6\varphi )$$

(36) $$\eta_{r}{\left[\frac{2\eta_{r}+5\lambda }{2+5\lambda }\right]}^{3/2}=1+\frac{5}{2}\varphi +\frac{9}{4}\left[\frac{1}{\psi \left(1+\frac{\psi }{2}\right){\left(1+\psi \right)}^{2}}\right]\;where\psi =2\left[\frac{1-{\left(\varphi /\varphi_{m}\right)}^{1/3}}{{\left(\varphi /\varphi_{m}\right)}^{1/3}}\right]$$

(37) $$\eta_{r}{\left[\frac{2\eta_{r}+5\lambda }{2+5\lambda }\right]}^{3/2}={\left(1-\varphi_{eff}\right)}^{-2.5}\;where\;\varphi_{eff}=\left[1+\left(\frac{1-\varphi_{m}}{\varphi_{m}}\right)\left(\sqrt{1-{\left(\frac{\varphi_{m}-\varphi }{\varphi_{m}}\right)}^{2}}\right)\right]\varphi$$

(38) $$\eta_{r}{\left[\frac{2\eta_{r}+5\lambda }{2+5\lambda }\right]}^{3/2}={\left(\frac{1-\varphi }{1-\frac{\varphi }{\varphi_{m}}}\right)}^{2.5\varphi_{m}/\left(1-\varphi_{m}\right)}$$

Equation (35) is referred to as model P11, Equation (36) as model P12, Equation (37) as model P13, and Equation (38) as model P14 in the remainder of this article. Note that in the limit λ→∞, models P11–P14 reduce to the corresponding models for suspensions: P11 reduces to the Thomas model [23], model P12 reduces to the Graham model [24], P13 reduces to the Pal model [25], and P14 reduces to the Brouwers model [19].

Table 2 gives a summary of all the emulsion viscosity models discussed in this section.

## 4. Comparisons of Model Predictions

**Figure 1:**
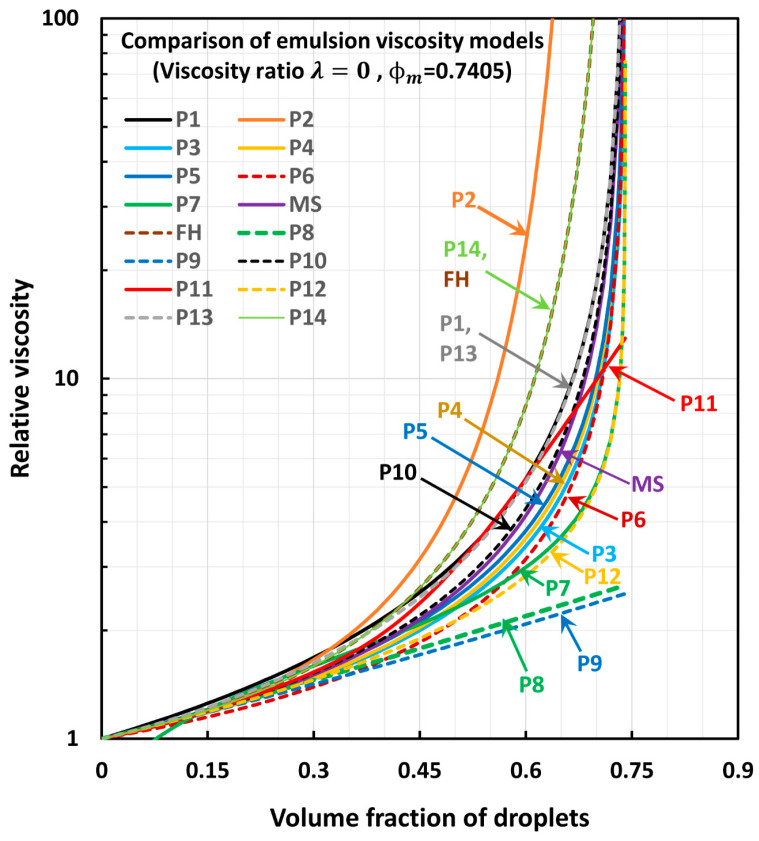
Comparison of model predictions of relative viscosity for λ=0.

**Figure 2:**
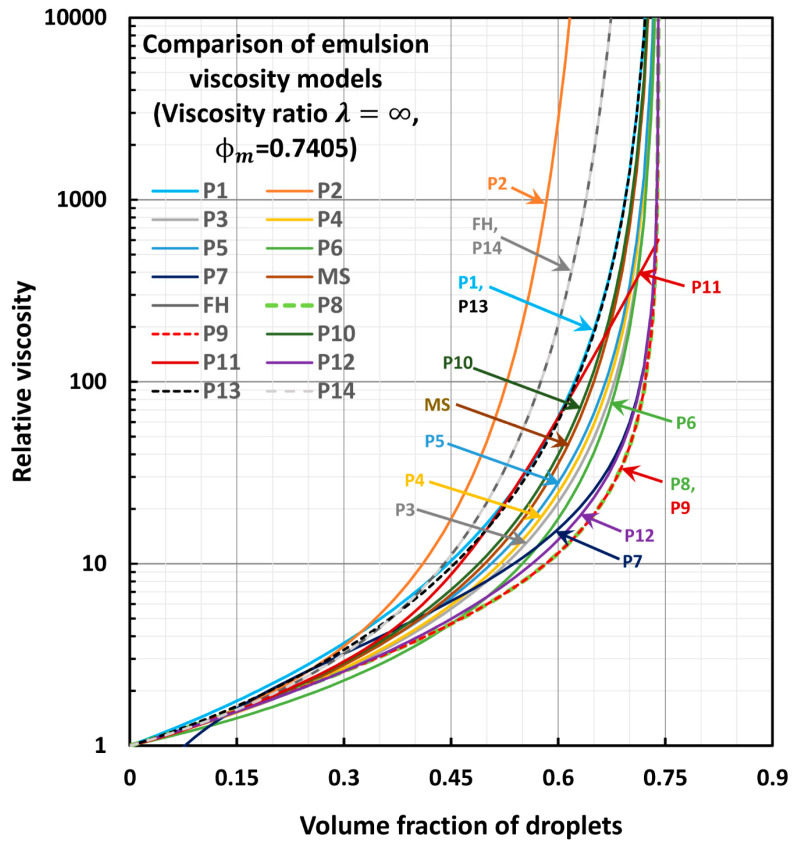
Comparison of model predictions of relative viscosity for λ=∞.

Figure 1 and Figure 2 compare the predictions of the models for two extreme values of viscosity ratio λ: λ=0 in Figure 1 and λ=∞ in Figure 2. The maximum packing volume fraction of droplets ($\varphi_{m})$ is taken to be 0.74048, corresponding to hexagonal close packing of uniform hard spheres. The comparisons reveal the following interesting information:

- When λ=0, all models other than the models P8, P9, and P11 diverge at $\varphi \to \varphi_{m}$. When λ=∞, only the model P11 does not diverge at $\varphi \to \varphi_{m}$. All other models diverge at the maximum packing volume fraction of particles.
- Models P3, P4, and P5 predict values very close to each other. The differences are slight, and the order is as follows: P5 > P4 > P3.
- Model P10 predictions fall slightly above those of model MS.
- Model P13 overlaps with model P1.
- Model P14 overlaps with model FH.
- Model P2 predicts the highest values of relative viscosity.
- Models P14 and FH predict the second highest values of relative viscosity.
- Models P8 and P9 give the lowest values of relative viscosity.
- Model P7 gives unrealistic values of relative viscosity at low values of φ (φ<0.12).
- At high values of concentration (φ>0.45), the order of predictions of models is generally as follows: P2 > P14 (=FH) > P1 (=P13) > P10 > MS > P5 > P4 > P3 > P6 > P7 > P12 > P8 > P9.

## 5. Comparisons of Model Predictions with Experimental Data

### 5.1. Experimental Data

Twenty-five sets of experimental data on the viscosity of stable emulsions were considered to evaluate the models. Table 3 summarizes the various experimental emulsion systems considered. The capillary number was small so that the deformation of droplets could be neglected. Both Newtonian and non-Newtonian emulsions were considered. The low-shear-rate viscosity was used for non-Newtonian emulsions. In some cases where the emulsions followed the power law behavior, the consistency index (equivalent to viscosity at a shear rate of 1 s^−1^) was used. Except for the nanoemulsions (sets 16–19), the emulsions consisted of non-Brownian droplets with droplet diameters in the micron range. The emulsions were monomodal in droplet size. Also, the nonhydrodynamic effects (other than Brownian motion) were negligible.

**Figure 3:**
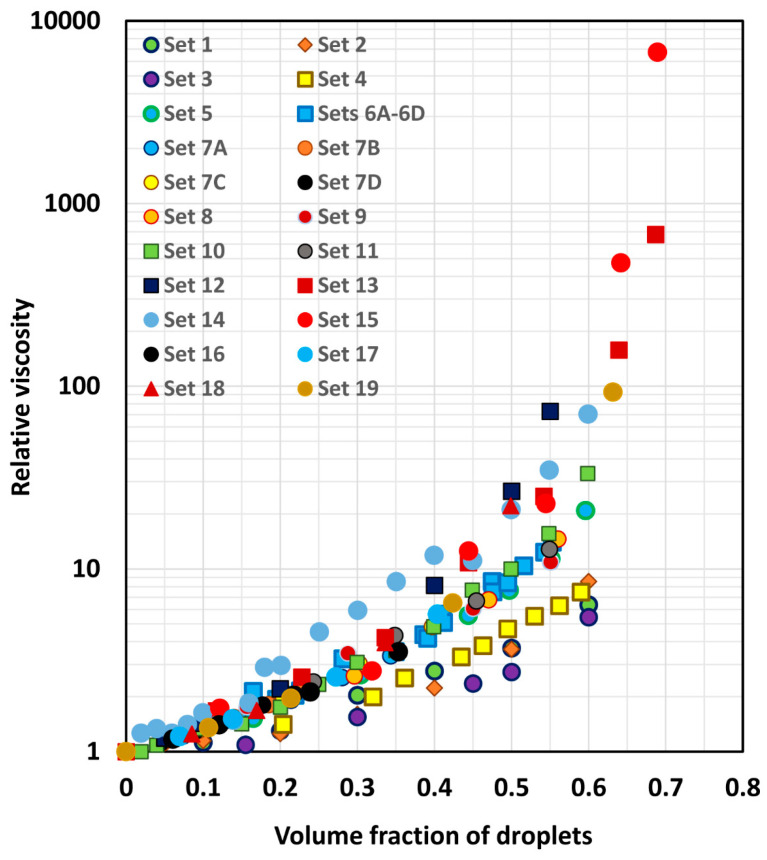
Experimental relative viscosity versus droplet volume fraction data collected from different sources.

Figure 3 shows the experimental relative viscosity versus volume fraction of the droplet data for all the sets considered. One can clearly see that the relative viscosity of different emulsion systems is different at any given volume fraction of droplets. The main reason of relative viscosity variation from one emulsion system to another is the viscosity ratio λ. The viscosity ratio λ is not the same for the different emulsion systems considered.

### 5.2. Maximum Packing Concentration of Droplets

The maximum packing concentration of droplets is estimated from the experimental relative viscosity versus volume fraction data at high values of φ (φ>0.30). When the data are plotted as $\eta_{r}^{-0.40}{\left[\frac{2\eta_{r}+5\lambda }{2+5\lambda }\right]}^{-0.6}$ versus φ, a linear relationship is observed. To estimate $\varphi_{m}$, this linear relationship is extended to $\eta_{r}^{-0.40}{\left[\frac{2\eta_{r}+5\lambda }{2+5\lambda }\right]}^{-0.6}=0$. Several authors [39,40] have used this approach to estimate $\varphi_{m}$ for suspensions.

**Figure 4:**
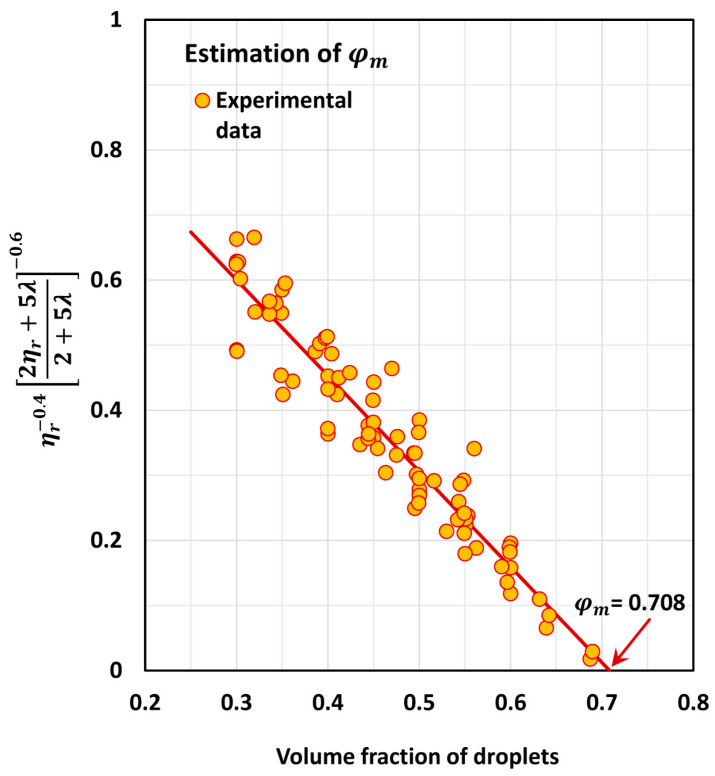
Estimation of maximum packing volume fraction of droplets.

Figure 4 shows the plot of $\eta_{r}^{-0.40}{\left[\frac{2\eta_{r}+5\lambda }{2+5\lambda }\right]}^{-0.6}$ versus φ data for emulsions. The experimental data for all sets of emulsions are plotted at high concentration (φ>0.30). The estimated value of $\varphi_{m}$ is 0.708. Note that $\varphi_{m}=0.637$ for the random packing of uniform spheres and $\varphi_{m}=0.74048$ for hexagonal packing of uniform spheres. Thus, the $\varphi_{m}$ value for the monomodal emulsions under consideration falls in between the random packing and hexagonal packing values.

### 5.3. Comparisons of Model Predictions with Experimental Data

**Figure 5:**
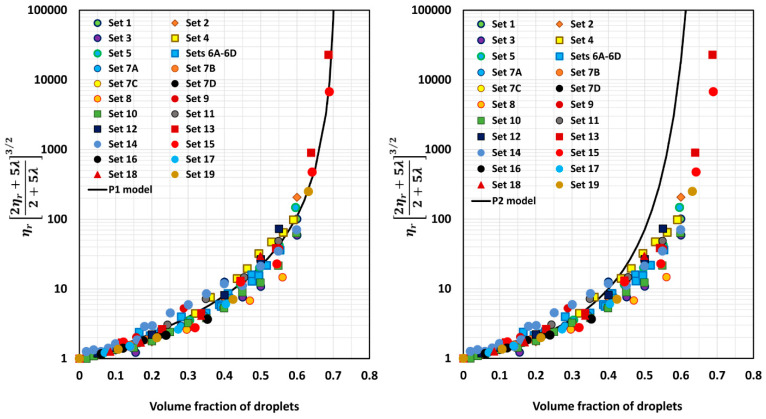
Comparisons of experimental data with predictions of models P1 and P2.

**Figure 6:**
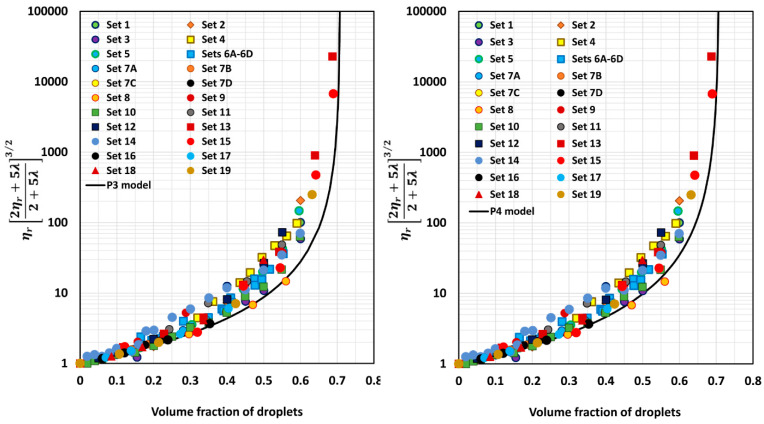
Comparisons of experimental data with predictions of models P3 and P4.

**Figure 7:**
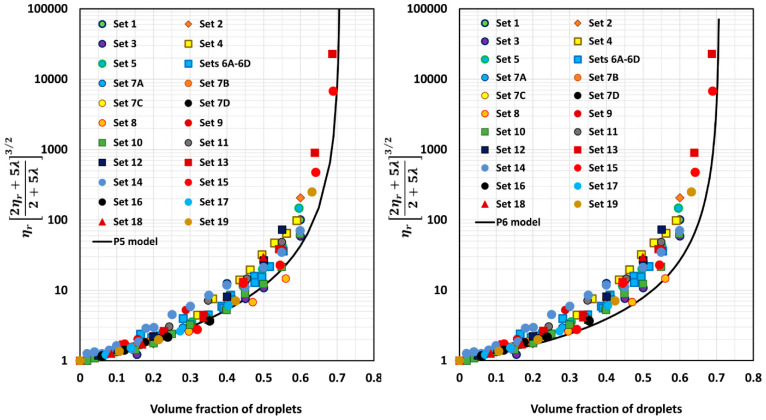
Comparisons of experimental data with predictions of models P5 and P6.

**Figure 8:**
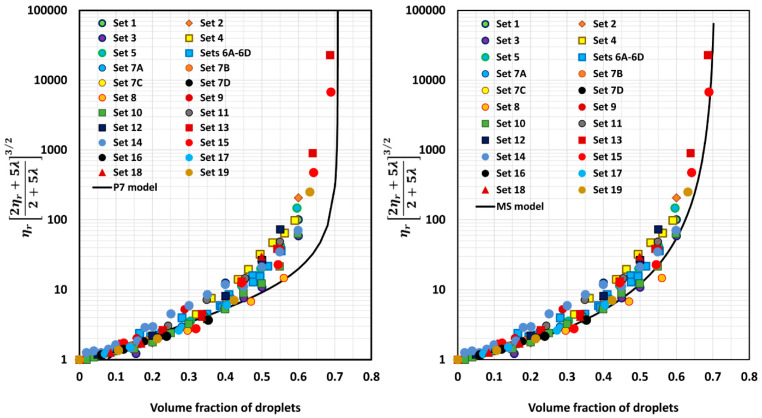
Comparisons of experimental data with predictions of models P7 and MS.

**Figure 9:**
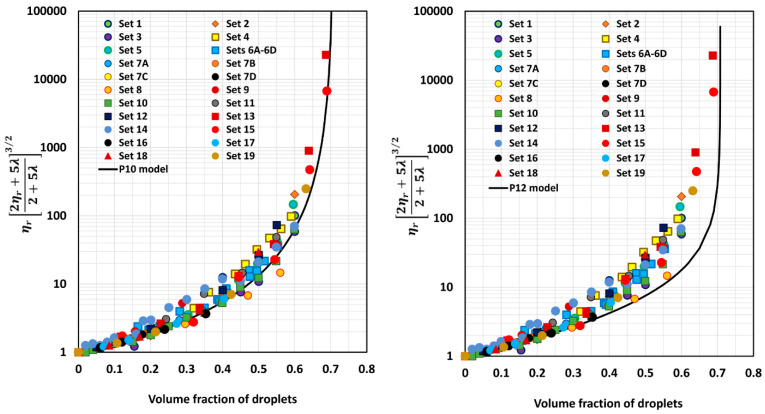
Comparisons of experimental data with predictions of models P10 and P12.

**Figure 10:**
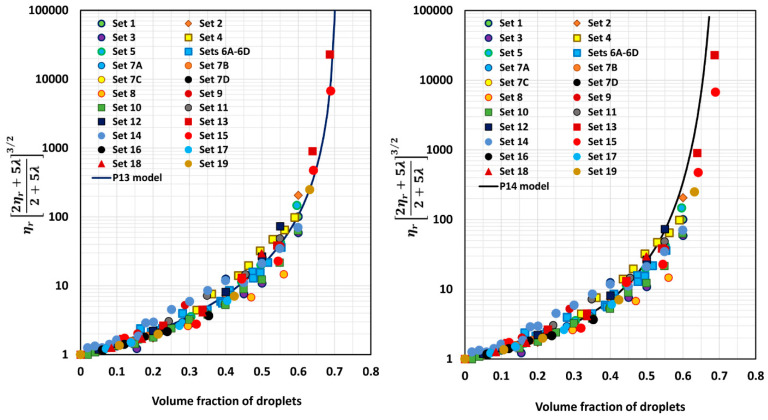
Comparisons of experimental data with predictions of models P13 and P14.

Figure 5, Figure 6, Figure 7, Figure 8, Figure 9 and Figure 10 show comparisons of model predictions with the experimental relative viscosity data for twenty-five sets of monomodal emulsions. The comparisons are shown for a majority of the models discussed in Section 3. The $\varphi_{m}\;$value used in model predictions is the estimated value of 0.708, as shown in Figure 4. It is interesting to note that the data for all different sets of emulsions fall on a single curve, confirming the validity of scaling the relative viscosity as $\eta_{r}{\left[\frac{2\eta_{r}+5\lambda }{2+5\lambda }\right]}^{3/2}$ versus φ basis, as predicted by the models. However, some models, such as P2 and P14, overpredict the relative viscosities, whereas the majority of the models (for example, P3–P7, MS, P10, and P12) underpredict the relative viscosities. Some models such as P1 and P13 describe the experimental data remarkably well.

The average percent relative error (*APRE*) is calculated for each model as follows:

(39) $$APRE=\frac{1}{n}\sum_{i=1}^{i=n}\frac{{\left(y_{exp}\right)}_{i}-{\left(y_{mod}\right)}_{i}}{{\left(y_{exp}\right)}_{i}}\times 100$$

where *n* is the total number of data points and $y=\eta_{r}{\left[\frac{2\eta_{r}+5\lambda }{2+5\lambda }\right]}^{3/2}$. The subscript “*exp*” indicates experimental value and the subscript “*mod*” indicates value predicted by the model. The *APRE* values of different models are summarized in Table 4. The models are listed in order of increasing *APRE*. Model P13 has the lowest *APRE* (3%) and hence it is the best model. Model P2 has the highest *APRE* (3.9 × 10^24^%) and, hence, it is the worst model in terms of predictability of the relative viscosity of emulsion. Model FH is equally bad in terms of predictability. It has an *APRE* of 1.3 × 10^8^%.

The deviations of model predictions are grouped into five different categories depending upon the absolute value of *APRE*. The categories are described below:

Absolute value of *APRE* ≤ 5%: Deviation is slight.
5% < Absolute value of *APRE* ≤ 10%: Deviation is moderate.
10% < Absolute value of *APRE* ≤ 30%: Deviation is substantial.
30% < Absolute value of *APRE* ≤ 100%: Deviation is severe.
Absolute value of *APRE* > %: Deviation is extreme.

Model P13 underpredicts the values only slightly (*APRE* = 3%); model P1 overpredicts moderately (*APRE* = −10.43%); model P11 underpredicts substantially (*APRE* = 12.28%); model P10 underpredicts substantially (APRE = 16.42%); model P5 underpredicts substantially (*APRE* = 19.81%); model MS underpredicts substantially (*APRE* = 20.24%); model P7 underpredicts substantially (*APRE* = 25.21%); model P4 underpredicts substantially (APRE = 26.35%); model P3 underpredicts substantially (*APRE* = 29.09%); model P12 underpredicts severely (*APRE* = 31.08%); model P8 underpredicts severely (*APRE* = 31.2%); model P9 underpredicts severely (*APRE* = 32%); model P6 underpredicts severely (*APRE* = 33.5%); model P14 overpredicts extremely (*APRE* = −381.75%); model FH overpredicts extremely (*APRE* = −1.3 × 10^8^%); and model P2 overpredicts extremely (*APRE* = −3.9 × 10^24^%).

## 6. The Effect of Modality of Droplet Size Distribution on Emulsion Viscosity

In the preceding sections, the discussion was related to the viscosity of concentrated monomodal or monodisperse emulsions. It is important from both practical and fundamental points of view to explore the influence of droplet size distribution on the viscosity of concentrated emulsions. As the concentration of dispersed phase (droplets) of emulsion is increased, the viscosity of the emulsion shoots up (see Figure 3). From a practical point of view, it is important to be able to formulate food emulsions with high concentration of dispersed phase but keeping the emulsion viscosity reasonable. One way to prevent the viscosity of a food emulsion from reaching unacceptable levels is to increase the modality of the emulsion droplet size distribution. Thus, it is important to explore the effect of modality of emulsion droplet size distribution on the viscosity of concentrated emulsions.

For the sake of simplicity, emulsions with a viscosity ratio of zero, that is, λ=0, were considered. As model P13 is the best model in terms of predictability of emulsion viscosity, this model was utilized to simulate the effect of modality of droplet size distribution on emulsion viscosity. In the limit λ→0, the model P13 reduces to the following expression:

(40) $$\eta_{r}={\left[1-\left\{1+\left(\frac{1-\varphi_{m}}{\varphi_{m}}\right)\left(\sqrt{1-{\left(\frac{\varphi_{m}-\varphi }{\varphi_{m}}\right)}^{2}}\right)\right\}\varphi \right]}^{-1}$$

**Figure 11:**
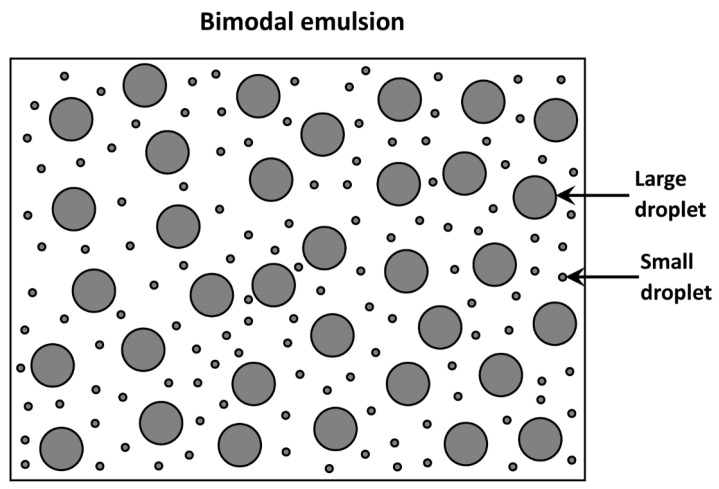
Bimodal emulsion of large and small droplets.

Consider a bimodal emulsion of two different size droplets: a coarse fraction of droplets with large uniform-size droplets and a fine fraction of droplets with small uniform-size droplets (see Figure 11). Let the volume fraction of fine droplets excluding the large droplets be $\varphi_{1}$, the volume fraction of large droplets in the emulsion be $\varphi_{2}$, and the overall concentration of all droplets be $\varphi_{T}$. Thus,

(41) $$\varphi_{1}=\frac{V_{1}}{V_{L}+V_{1}};\;\varphi_{2}=\frac{V_{2}}{V_{L}+V_{1}+V_{2}};\;\varphi_{T}=\frac{V_{1}+V_{2}}{V_{L\;}+V_{1}+V_{2}}$$

where $V_{L}$, $V_{1}$, and $V_{2}$ are the volumes of the suspending medium (matrix liquid), fine droplets, and large droplets, respectively.

As the fine droplets are very small compared with the large droplets, the fine-droplet emulsion (fine droplets together with suspending medium) can be treated as an effective homogeneous medium with respect to large droplets. Consequently,

(42) $$\frac{\eta_{overall-emulsion}}{\eta_{fine-emulsion}}=H\left(\varphi_{2}\right)$$

(43) $$\frac{\eta_{fine-emulsion}}{\eta_{m}}=H\left(\varphi_{1}\right)$$

where $\eta_{overall-emulsion}$ is the viscosity of the whole emulsion, $\eta_{fine-emulsion}$ is the viscosity of the fine emulsion (fine droplets together with suspending medium), $\eta_{m}$ is the matrix liquid viscosity, and $H\left(\varphi \right)$ is the relative viscosity function of the monomodal emulsion given by Equation (40). From Equations (42) and (43), it follows that the relative viscosity of the whole emulsion is:

(44) $$\eta_{r}=\frac{\eta_{overall-emulsion}}{\eta_{m}}=\frac{\eta_{overall-emulsion}}{\eta_{fine-emulsion}}\times \frac{\eta_{fine-emulsion}}{\eta_{m}}=H\left(\varphi_{1}\right)H\left(\varphi_{2}\right)$$

Combining Equations (40) and (44), we obtain the following expression for the relative viscosity of a bimodal emulsion:

(45) $$\eta_{r}={\left[1-\left\{1+\left(\frac{1-\varphi_{m}}{\varphi_{m}}\right)\left(\sqrt{1-{\left(\frac{\varphi_{m}-\varphi_{1}}{\varphi_{m}}\right)}^{2}}\right)\right\}\varphi_{1}\right]}^{-1}{\left[1-\left\{1+\left(\frac{1-\varphi_{m}}{\varphi_{m}}\right)\left(\sqrt{1-{\left(\frac{\varphi_{m}-\varphi_{2}}{\varphi_{m}}\right)}^{2}}\right)\right\}\varphi_{2}\right]}^{-1}$$

Given the overall concentration of all droplets $\varphi_{T}$ and fraction $f_{c}$ of coarse droplets in a mixture of coarse and fine droplets, it can be readily shown that:

(46) $$\varphi_{2}=f_{c}\varphi_{T}$$

(47) $$\varphi_{1}=\frac{\varphi_{T}-\varphi_{2}}{1-\varphi_{2}}$$

Note that:

(48) $$f_{c}=\frac{V_{2}}{V_{1}+V_{2}}$$

Thus, the relative viscosity of a bimodal emulsion can be estimated from Equation (45) for any given overall concentration of droplets $\varphi_{T}$ and fraction of coarse droplets $f_{c}$ in a mixture of fine and coarse droplets. 

**Figure 12:**
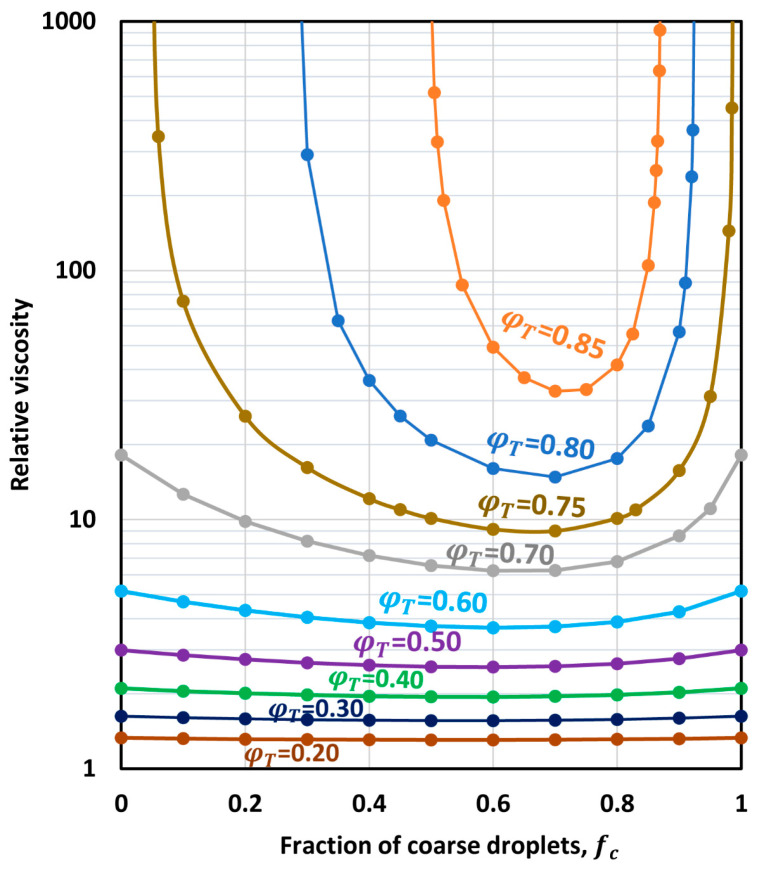
Relative viscosity of bimodal emulsions as a function of fraction of coarse droplets for different overall droplet volume fractions.

Figure 12 shows the relative viscosities of bimodal emulsions for various values of overall droplet concentration $\varphi_{T}$. The data are plotted as relative viscosity versus fraction of coarse droplets in a mixture of fine and coarse droplets. The plots were generated from Equation (45). The maximum packing volume fraction of monomodal emulsion ($\varphi_{m}$) was taken to be 0.74048 corresponding to hexagonal close packing of uniform spheres.

As can be seen in Figure 12, a large drop in the relative viscosity of the emulsion occurs when a monomodal emulsion is replaced by a bimodal emulsion at the same overall droplet concentration $\varphi_{T}$. The effect of droplet size modality is especially large when $\varphi_{T}\geq 0.75$. It should also be noted that the relative viscosity of a bimodal emulsion exhibits a minimum at some fraction of coarse droplets $f_{c}$.

**Figure 13:**
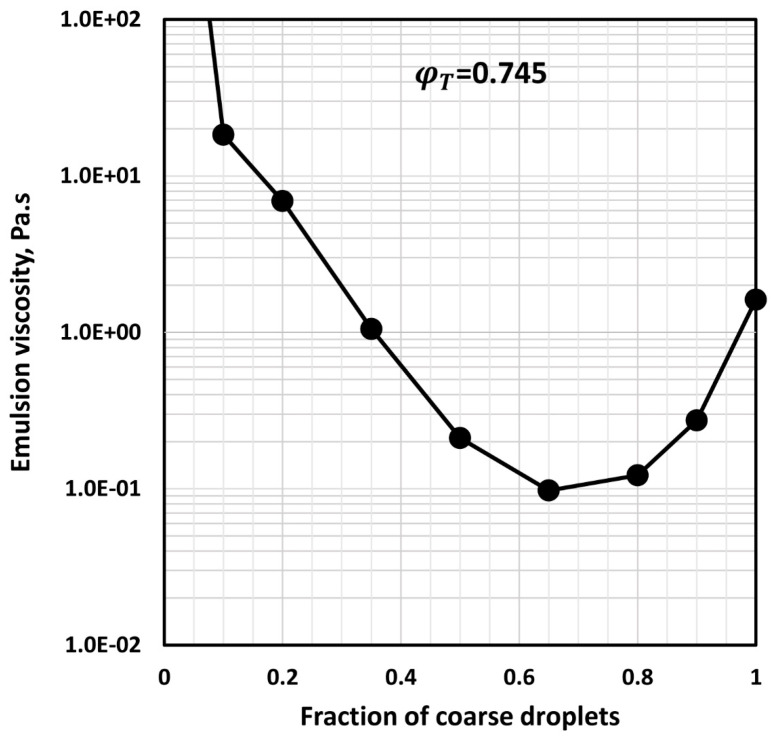
Low-shear viscosity of bimodal oil-in-water emulsion as a function of fraction of coarse droplets.

The minimum viscosity of concentrated bimodal emulsion was confirmed through experiments. For example, Figure 13 shows the experimental data of Pal [41] for a bimodal oil-in-water emulsion. A minimum in low-shear viscosity occurs at a coarse droplet fraction of around 0.65.

## 7. The Effect of Capillary Number on the Viscosity of Concentrated Emulsions

In the processing and pumping of food emulsions, the capillary number is not zero. Thus, it is important to investigate the influence of capillary number on the viscosity of emulsions. As demonstrated by Frankel and Acrivos [10] in their equation, Equation (7), the relative viscosity of emulsions is a function of Ca. The emulsions exhibit shear-thinning behavior with the increase in Ca due to the orientation and elongation of droplets in the direction of flow. However, the Frankel and Acrivos equation, Equation (7), is valid for infinitely dilute emulsions (φ→0).

Only a few analytical studies have been published exploring the influence of capillary number on the viscosity of concentrated emulsions. Using the analogy between shear modulus and shear viscosity and applying the differential effective medium scheme, Pal [42] developed three relative viscosity models for concentrated monodisperse emulsions applicable at any capillary number. The Pal models are as follows:

(49) $$\eta_{r}{\left[\frac{M-P+32\eta_{r}}{M-P+32}\right]}^{R-1.25}{\left[\frac{M+P-32}{M+P-32\eta_{r}}\right]}^{R+1.25}={\left(1-\varphi \right)}^{-2.5}$$

(50) $$\eta_{r}{\left[\frac{M-P+32\eta_{r}}{M-P+32}\right]}^{R-1.25}{\left[\frac{M+P-32}{M+P-32\eta_{r}}\right]}^{R+1.25}=exp\left(\frac{2.5\varphi }{1-\frac{\varphi }{\varphi_{m}}}\right)$$

(51) $$\eta_{r}{\left[\frac{M-P+32\eta_{r}}{M-P+32}\right]}^{R-1.25}{\left[\frac{M+P-32}{M+P-32\eta_{r}}\right]}^{R+1.25}={\left(1-\frac{\varphi }{\varphi_{m}}\right)}^{-2.5\varphi_{m}}$$

where

(52) $$M=\sqrt{\left(64/{Ca}^{2}\right)+1225\lambda^{2}+1232\left(\lambda /Ca\right)}$$

(53) $$P=\left(8/Ca\right)-3\lambda$$

(54) $$R=\frac{\left(22/Ca\right)+43.75\lambda }{\sqrt{\left(64/{Ca}^{2}\right)+1225\lambda^{2}+1232\left(\lambda /Ca\right)}}$$

Faroughi and Huber [18] also investigated the effect of capillary number on the relative viscosity of concentrated monodisperse emulsions. Their model is given as:

(55) $$\eta_{r}{\left[\frac{N+Wk{Ca}^{2}\eta_{r}^{2}}{N+Wk{Ca}^{2}}\right]}^{0.5\left(\frac{N}{W}-1\right)}={\left[\frac{\varphi_{m}-\varphi }{\varphi_{m}(1-\varphi )}\right]}^{-N\varphi_{m}/\left(1-\varphi_{m}\right)}$$

where

(56) $$W=\frac{140\left(\lambda^{3}+\lambda^{2}-\lambda -1\right)}{28\left(2\lambda +3\right){\left(\lambda +1\right)}^{2}}\;;\;k={\left[\frac{\left(2\lambda +3\right)\left(19\lambda +16\right)}{40\left(\lambda +1\right)}\right]}^{2};\;N=\left(\frac{2+5\lambda }{2+2\lambda }\right)$$

Faroughi and Huber [18] applied the differential effective medium scheme incorrectly to derive their model (Equation (55)). They assumed that the viscosity ratio is constant during the integration process. This is an invalid assumption as viscosity ratio varies during successive additions of differential amounts of dispersed phase (droplets) to the emulsion in the process of developing the equation for concentrated emulsion. Pal [15,42] applied the differential effective scheme correctly in the derivation of his equations for concentrated emulsions. Thus, the Faroughi and Huber model, Equation (55), is of questionable validity.

The models expressed in Equations (49)–(51) can be re-written in a general form as:

(57) $$\eta_{r}{\left[\frac{M-P+32\eta_{r}}{M-P+32}\right]}^{R-1.25}{\left[\frac{M+P-32}{M+P-32\eta_{r}}\right]}^{R+1.25}=f\left(\varphi ,\varphi_{m}\right)$$

In the limit Ca→0, Equation (57) reduces to the form of the models discussed in Section 3 (see Table 2) as:

(58) $$\eta_{r}{\left[\frac{2\eta_{r}+5\lambda }{2+5\lambda }\right]}^{3/2}=f\left(\varphi ,\varphi_{m}\right)$$

Thus, the models valid for Ca→0 (discussed in Section 3) can be generalized to non-zero capillary numbers by replacing the left-hand side of Equation (58) with the left-hand side of Equation (57).

As the best model valid in the limit Ca→0 is found to be model P13 (Equation (37)), the following generalized version of P13 is proposed as a new model for concentrated emulsions applicable at any Ca:

(59) $$\eta_{r}{\left[\frac{M-P+32\eta_{r}}{M-P+32}\right]}^{R-1.25}{\left[\frac{M+P-32}{M+P-32\eta_{r}}\right]}^{R+1.25}={\left[1-\left\{1+\left(\frac{1-\varphi_{m}}{\varphi_{m}}\right)\left(\sqrt{1-{\left(\frac{\varphi_{m}-\varphi }{\varphi_{m}}\right)}^{2}}\right)\right\}\varphi \right]}^{-2.5}$$

**Figure 14:**
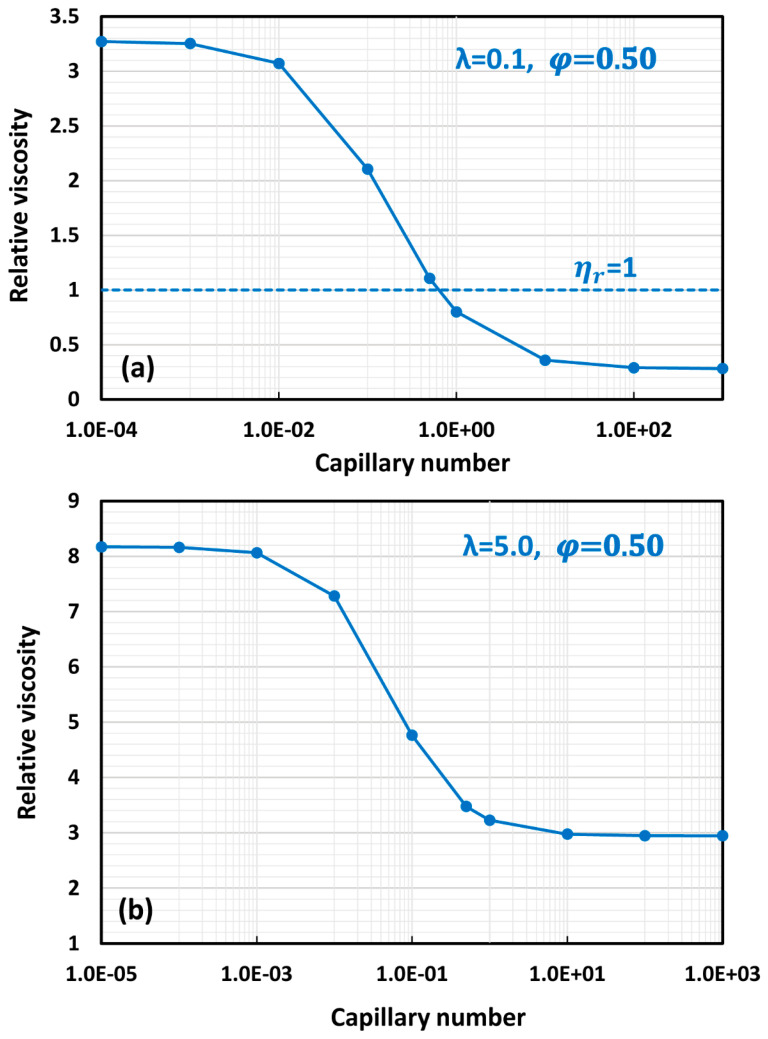
Relative viscosity versus capillary number plots generated from the proposed model, Equation (59), for (**a**) *λ* = 0.1 and (**b**) *λ* = 5.0.

Figure 14 shows the relative viscosity versus capillary number plots for concentrated emulsions at two different values of viscosity ratio (λ) generated from the proposed model Equation (59). The value of $\varphi_{m}$ used in the model calculations is 0.74048, corresponding to hexagonal close packing of uniform spheres. As expected, emulsions exhibit shear-thinning non-Newtonian behavior. The decrease in viscosity with the increase in capillary number is due to the orientation and elongation of droplets in the direction of flow. At a high value of viscosity ratio (*λ* = 5), the relative viscosity of the emulsion is always greater than unity regardless of the capillary number. However, at a small value of viscosity ratio (*λ* = 0.1), the relative viscosity becomes less than unity at high values of capillary number.

**Figure 15:**
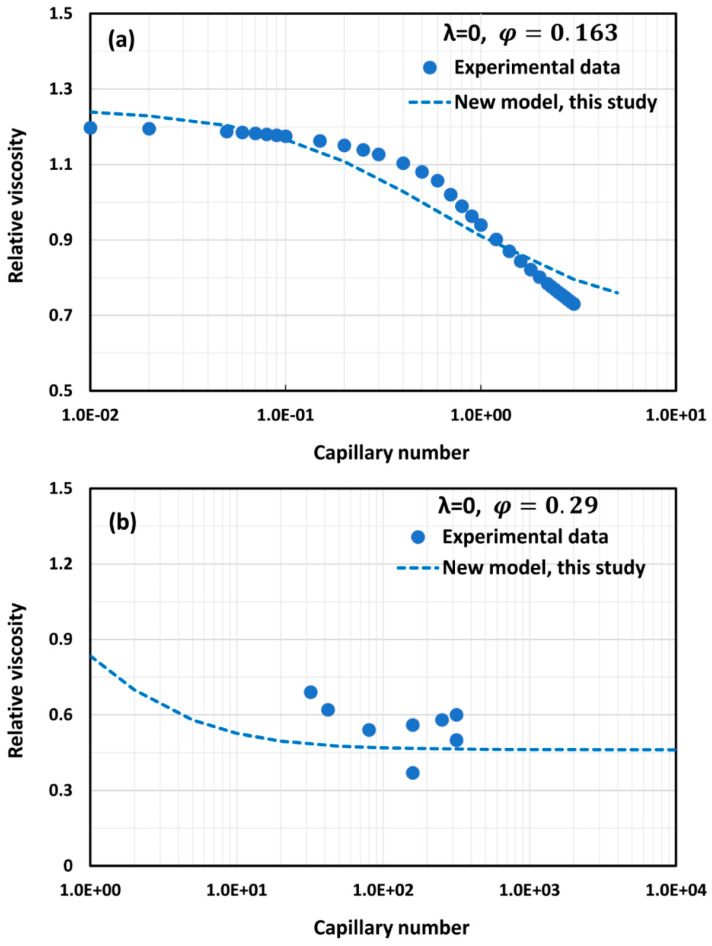
Comparisons of model (Equation (59)) predictions with experimental data: (**a**) experimental data of Rust and Manga [43] and (**b**) experimental data of Stein and Spera [44].

**Figure 16:**
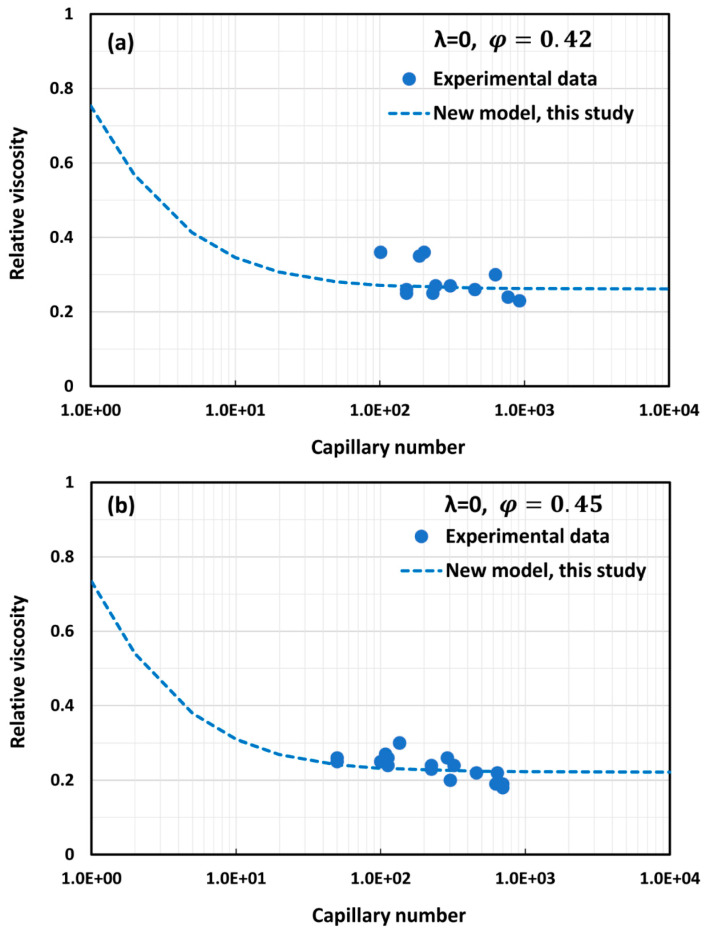
Comparisons of model (Equation (59)) predictions with experimental data of Stein and Spera [44] at volume fractions of (**a**) φ=0.42 and (**b**) φ=0.45.

Only a limited amount of experimental data are available on the effect of capillary number on the relative viscosity of emulsions. The available experimental data for the most part are restricted to emulsions with λ = 0. Figure 15 and Figure 16 show comparisons of the model (Equation (59)) predictions with the available experimental data. The model predictions with $\varphi_{m}=0.74048$ show good agreement with the experimental data.

## 8. Conclusions

Herein, the models describing relative viscosity versus the volume fraction of droplets for concentrated monodisperse emulsions published in the twenty-first century were reviewed, compared, and evaluated using a large body of experimental viscosity data available on emulsions. Based on the average percent relative error (*APRE*), the models were rated. The worst performer was model P2 (Equation (17)) with an extremely large *APRE* ($\left|APRE\right|=3.9\times 10^{24}\%$). The best perfomer was model P13 (Equation (37)) with a negligible *APRE* ($\left|APRE\right|\;\leq 3\;\%$). The best model identified on the basis of *APRE*, that is, model P13, was used to simulate the effect of droplet size distribution on emulsion viscosity. A large drop in the relative viscosity of the emulsion occurred when a monomodal emulsion was replaced by a bimodal emulsion at the same droplet concentration. The effect of droplet size modality on emulsion viscosity increased with the increase in droplet concentration.

Finally, the influence of capillary number on the viscosity of concentrated emulsions was discussed. A new generalized version of model P13 (identified as the best model, Equation (37)) was proposed and evaluated for the influence of capillary number on emulsion viscosity. The models discussed and developed in this article could be used as a tool to design efficient food processing operations and to formulate food emulsions with the desired rheological properties.

## Figures and Tables

**Table 1:** Examples of emulsion-type foods.

Food Type	Emulsion Type	Inclusions	Suspending Medium	Volume Percent of Inclusions
Milk	O/W	Oil globules	Aqueous phase	3 to 4
Butter	W/O	Water globules	Oil and fat crystals	About 15
Margarine	W/O	Water globules	Oil and fat crystals	15 to 50
Coffee whiteners	O/W	Oil globules	Aqueous phase	10 to 15
Salad dressings	O/W	Oil globules	Aqueous phase	Larger than 30

**Table 2:** Summary of the various emulsion viscosity models.

Model Symbol	Equation Number	Model Description	Reference and Year Published
P1	Equation (15)	$\eta_{r}{\left[\frac{2\eta_{r}+5\lambda }{2+5\lambda }\right]}^{3/2}={\left(1-\frac{\varphi }{\varphi_{m}}\right)}^{-2.5}$	Pal, 2000 [14]
P2	Equation (17)	$\eta_{r}{\left[\frac{2\eta_{r}+5\lambda }{2+5\lambda }\right]}^{3/2}=exp\left(\frac{2.5\varphi }{1-\varphi /\varphi_{m}}\right)$	Pal, 2001 [15]
P3	Equation (19)	$\eta_{r}{\left[\frac{2\eta_{r}+5\lambda }{2+5\lambda }\right]}^{3/2}={\left(1-\frac{\varphi }{\varphi_{m}}\right)}^{-2.5\varphi_{m}}$	Pal, 2001 [15]
P4	Equation (22)	$\eta_{r}{\left[\frac{2\eta_{r}+5\lambda }{2+5\lambda }\right]}^{3/2}={\left(1+\frac{1.25\varphi }{1-\frac{\varphi }{\varphi_{m}}}\right)}^{2}$	Pal, 2001 [16]
P5	Equation (23)	$\eta_{r}{\left[\frac{2\eta_{r}+5\lambda }{2+5\lambda }\right]}^{3/2}={\left(1-\frac{\varphi }{\varphi_{m}}\right)}^{-2}$	Pal, 2001 [16]
P6	Equation (24)	$\eta_{r}{\left[\frac{2\eta_{r}+5\lambda }{2+5\lambda }\right]}^{3/2}={\left(1+\frac{0.75\left(\varphi /\varphi_{m}\right)}{1-\frac{\varphi }{\varphi_{m}}}\right)}^{2}$	Pal, 2001 [16]
P7	Equation (25)	$\eta_{r}{\left[\frac{2\eta_{r}+5\lambda }{2+5\lambda }\right]}^{3/2}=\frac{9}{8}\left[\frac{{\left(\varphi /\varphi_{m}\right)}^{1/3}}{1-{\left(\varphi /\varphi_{m}\right)}^{1/3}}\right]$ $\left(\varphi \geq 0.1042\varphi_{m}\right)$	Pal, 2001 [16]
MS	Equation (26)	$\eta_{r}{\left[\frac{2\eta_{r}+5\lambda }{2+5\lambda }\right]}^{3/2}={\left(1-\frac{\varphi }{1-c\varphi }\right)}^{-2.5}$ where $c=\frac{1-\varphi_{m}}{\varphi_{m}}$	Mendoza and Santamaria-Holek, 2010 [17]
FH	Equation (27)	${\eta_{r}=\left[\frac{\varphi_{m}-\varphi }{\varphi_{m}(1-\varphi )}\right]}^{-N\varphi_{m}/\left(1-\varphi_{m}\right)}$ where $N=\left(\frac{2+5\lambda }{2+2\lambda }\right)$	Faroughi and Huber, 2015 [18]
P8	Equation (28)	$\eta_{r}=1+\frac{5}{2}\left[\frac{\varphi_{eff}\left(\frac{2+5\lambda }{5+5\lambda }\right)}{1-\varphi_{eff}\left(\frac{2+5\lambda }{5+5\lambda }\right)}\right]$ where: $\varphi_{eff}=\left[1+\left(\frac{1-\varphi_{m}}{\varphi_{m}}\right)\left(\sqrt{1-{\left(\frac{\varphi_{m}-\varphi }{\varphi_{m}}\right)}^{2}}\right)\right]\varphi$	Pal, 2016 [20]
P9	Equation (30)	$\eta_{r}=1+\frac{5}{2}\left(\frac{\varphi_{eff}}{1-\varphi_{eff}}\right)$ where: $\varphi_{eff}=\left[1+\left(\frac{1-\varphi_{m}}{\varphi_{m}}\right)\left(\sqrt{1-{\left(\frac{\varphi_{m}-\varphi^{*}}{\varphi_{m}}\right)}^{2}}\right)\right]\varphi^{*}$ $\varphi^{*}=\left(\frac{2+5\lambda }{5+5\lambda }\right)\varphi$	Pal, 2017 [21]
P10	Equation (33)	$\eta_{r}{\left[\frac{2\eta_{r}+5\lambda }{2+5\lambda }\right]}^{3/2}={\left(1-\varphi_{eff}\right)}^{-2.5}$ where $\varphi_{eff}=\left\{1+\left[\frac{1-\varphi_{m}}{\varphi_{m}^{2}}\right]\varphi \right\}\varphi$	Pal, 2020 [22]
P11	Equation (35)	$\eta_{r}{\left[\frac{2\eta_{r}+5\lambda }{2+5\lambda }\right]}^{3/2}=1+2.5\varphi +10.05\varphi^{2}+0.00273exp(16.6\varphi )$	This study
P12	Equation (36)	$\eta_{r}{\left[\frac{2\eta_{r}+5\lambda }{2+5\lambda }\right]}^{3/2}=1+\frac{5}{2}\varphi +\frac{9}{4}\left[\frac{1}{\psi \left(1+\frac{\psi }{2}\right){\left(1+\psi \right)}^{2}}\right]$ where $\psi =2\left[\frac{1-{\left(\varphi /\varphi_{m}\right)}^{1/3}}{{\left(\varphi /\varphi_{m}\right)}^{1/3}}\right]$	This study
P13	Equation (37)	$\eta_{r}{\left[\frac{2\eta_{r}+5\lambda }{2+5\lambda }\right]}^{3/2}={\left(1-\varphi_{eff}\right)}^{-2.5}$ where: $\varphi_{eff}=\left[1+\left(\frac{1-\varphi_{m}}{\varphi_{m}}\right)\left(\sqrt{1-{\left(\frac{\varphi_{m}-\varphi }{\varphi_{m}}\right)}^{2}}\right)\right]\varphi$	This study
P14	Equation (38)	$\eta_{r}{\left[\frac{2\eta_{r}+5\lambda }{2+5\lambda }\right]}^{3/2}={\left(\frac{1-\varphi }{1-\frac{\varphi }{\varphi_{m}}}\right)}^{2.5\varphi_{m}/\left(1-\varphi_{m}\right)}$	This study

**Table 3:** Details of experimental emulsion systems used in the evaluation of the viscosity models (25 sets).

Set No	Emulsion Type	Range of *ϕ*	Viscosity Ratio (*λ*)	Description	Source
1	Oil-in-water	0–0.60	4.15 × 10^−3^	Emulsions thickened by polymer; odourless kerosine as the oil phase	Pal [26]
2	Oil-in-water	0–0.60	1.12 × 10^−2^	Emulsions thickened by polymer; odourless kerosine as the oil phase	
3	Oil-in-water	0–0.60	5.82 × 10^−2^	Emulsions thickened by polymer; petroleum oil (EDM) as the oil phase	Pal [27]
4	Water-in-oil	0–0.65	1.65 × 10^−1^	Emulsions prepared from white mineral oil (Bayol-35)	Hsieh [28]
5	Oil-in-water	0–0.596	2.574	Emulsions prepared from white mineral oil (Bayol-35)	Pal [29]
6A	Oil-in-water	0–0.516	5.52	The droplet sizes of these emulsions were different, but they were prepared from the same oil and aqueous phase; petroleum oil (EDM) as the oil phase	Pal [30]
6B	Oil-in-water	0–0.494			
6C	Oil-in-water	0–0.553			
6D	Oil-in-water	0–0.543			
7A	Oil-in-water	0–0.343	5.573	These were emulsions of milk fat. The suspending mediums of emulsions were skim milk, dilute and concentrated skim milk	Leviton and Leighton [31]
7B	Oil-in-water	0–0.230	12.35		
7C	Oil-in-water	0–0.397	21.74		
7D	Oil-in-water	0–0.218	29.41		
8	Oil-in-water	0–0.635	1.17 × 10^3^	These were emulsions of heavy oils	Pal [15]
9	Oil-in-water	0–0.551	2.67	Emulsions prepared from white mineral oil (Bayol-35)	Pal [29]
10	Oil-in-water	0–0.60	23.7	Emulsions prepared from food-grade white mineral oil (Purity FG W/O-15)	Bains [32]
11	Oil-in-water	0–0.549	2.91	Emulsions prepared from white mineral oil (Bayol-35)	Buhidma [33]
12	Oil-in-water	0–0.55	Droplets treated as solid particles	Pickering emulsions: droplets coated with a layer of solid nanoparticles; oil phase as a mixture of equal volumes of isopropyl myristate and dodecane	Wolf et al. [34]
13	Oil-in-water	0–0.687	28.26	Emulsions prepared from food-grade white mineral oil (Purity FG W/O-15)	Pal and Pal [35]
14	Oil-in-water	0–0.599	Droplets treated as solid particles	Pickering emulsions: droplets coated with starch nanoparticles; food-grade white mineral oil (Purity FG W/O-15) as the oil phase	Bains and Pal [36]
15	Oil-in-water	0–0.689	Droplets treated as solid particles	Pickering emulsions: droplets coated with cellulose nanocrystals; food-grade white mineral oil (Purity FG W/O-15) as the oil phase	Kinra and Pal [37]
16	Oil-in-water	0–0.353	39	These were nanoemulsions consisting of solvated droplets prepared using different concentrations of emulsifier. The oil was medicinal oil. The droplet diameters were as follows: Set 16 (205 nm), Set 17 (102 nm), Set 18 (58.5 nm), and Set 19 (27.5 nm)	Van der Waarden [38]
17	Oil-in-water	0–0.404			
18	Oil-in-water	0–0.499			
19	Oil-in-water	0–0.631			

**Table 4:** APRE values of different models.

Model Symbol, Equation Number, and Reference	Average Percent Relative Error (*APRE*)	Model Description	Comments
P13, Equation (37), new model, this study	3%	$\eta_{r}{\left[\frac{2\eta_{r}+5\lambda }{2+5\lambda }\right]}^{3/2}={\left(1-\varphi_{eff}\right)}^{-2.5}$ where: $\varphi_{eff}=\left[1+\left(\frac{1-\varphi_{m}}{\varphi_{m}}\right)\left(\sqrt{1-{\left(\frac{\varphi_{m}-\varphi }{\varphi_{m}}\right)}^{2}}\right)\right]\varphi$	Best model. Model underpredicts only slightly
P1, Equation (15), Pal [14]	−10.43%	$\eta_{r}{\left[\frac{2\eta_{r}+5\lambda }{2+5\lambda }\right]}^{3/2}={\left(1-\frac{\varphi }{\varphi_{m}}\right)}^{-2.5}$	Model overpredicts moderately
P11, Equation (35), new model, this study	12.28%	$\eta_{r}{\left[\frac{2\eta_{r}+5\lambda }{2+5\lambda }\right]}^{3/2}=1+2.5\varphi +10.05\varphi^{2}+0.00273exp(16.6\varphi )$	Model underpredicts substantially
P10, Equation (33), Pal [22]	16.42%	$\eta_{r}{\left[\frac{2\eta_{r}+5\lambda }{2+5\lambda }\right]}^{3/2}={\left(1-\varphi_{eff}\right)}^{-2.5}$ where $\varphi_{eff}=\left\{1+\left[\frac{1-\varphi_{m}}{\varphi_{m}^{2}}\right]\varphi \right\}\varphi$	Model underpredicts substantially
P5, Equation (23), Pal [16]	19.81%	$\eta_{r}{\left[\frac{2\eta_{r}+5\lambda }{2+5\lambda }\right]}^{3/2}={\left(1-\frac{\varphi }{\varphi_{m}}\right)}^{-2}$	Model underpredicts substantially
MS, Equation (26), Mendoza and Santamaria-Holek [17]	20.24%	$\eta_{r}{\left[\frac{2\eta_{r}+5\lambda }{2+5\lambda }\right]}^{3/2}={\left(1-\frac{\varphi }{1-c\varphi }\right)}^{-2.5}$ where $c=\frac{1-\varphi_{m}}{\varphi_{m}}$	Model underpredicts substantially
P7, Equation (25), Pal [16]	25.21%	$\eta_{r}{\left[\frac{2\eta_{r}+5\lambda }{2+5\lambda }\right]}^{3/2}=\frac{9}{8}\left[\frac{{\left(\varphi /\varphi_{m}\right)}^{1/3}}{1-{\left(\varphi /\varphi_{m}\right)}^{1/3}}\right]$ $\left(\varphi \geq 0.1042\varphi_{m}\right)$	Model underpredicts substantially
P4, Equation (22), Pal [16]	26.35%	$\eta_{r}{\left[\frac{2\eta_{r}+5\lambda }{2+5\lambda }\right]}^{3/2}={\left(1+\frac{1.25\varphi }{1-\frac{\varphi }{\varphi_{m}}}\right)}^{2}$	Model underpredicts substantially
P3, Equation (19), Pal [15]	29.09%	$\eta_{r}{\left[\frac{2\eta_{r}+5\lambda }{2+5\lambda }\right]}^{3/2}={\left(1-\frac{\varphi }{\varphi_{m}}\right)}^{-2.5\varphi_{m}}$	Model underpredicts substantially
P12, Equation (36), new model, this study	31.08%	$\;\;\eta_{r}{\left[\frac{2\eta_{r}+5\lambda }{2+5\lambda }\right]}^{3/2}=1+\frac{5}{2}\varphi +\frac{9}{4}\left[\frac{1}{\psi \left(1+\frac{\psi }{2}\right){\left(1+\psi \right)}^{2}}\right]$ where $\psi =2\left[\frac{1-{\left(\varphi /\varphi_{m}\right)}^{1/3}}{{\left(\varphi /\varphi_{m}\right)}^{1/3}}\right]$	Model underpredicts severely
P8, Equation (28), Pal [20]	31.2%	$\eta_{r}=1+\frac{5}{2}\left[\frac{\varphi_{eff}\left(\frac{2+5\lambda }{5+5\lambda }\right)}{1-\varphi_{eff}\left(\frac{2+5\lambda }{5+5\lambda }\right)}\right]$ where: $\varphi_{eff}=\left[1+\left(\frac{1-\varphi_{m}}{\varphi_{m}}\right)\left(\sqrt{1-{\left(\frac{\varphi_{m}-\varphi }{\varphi_{m}}\right)}^{2}}\right)\right]\varphi$	Model underpredicts severely
P9, Equation (30), Pal [21]	32%	$\eta_{r}=1+\frac{5}{2}\left(\frac{\varphi_{eff}}{1-\varphi_{eff}}\right)$ where: $\varphi_{eff}=\left[1+\left(\frac{1-\varphi_{m}}{\varphi_{m}}\right)\left(\sqrt{1-{\left(\frac{\varphi_{m-}\varphi^{*}}{\varphi_{m}}\right)}^{2}}\right)\right]\varphi^{*}$ Note that: $\varphi^{*}=\left(\frac{2+5\lambda }{5+5\lambda }\right)\varphi$	Model underpredicts severely
P6, Equation (24), Pal [16]	33.5%	$\eta_{r}{\left[\frac{2\eta_{r}+5\lambda }{2+5\lambda }\right]}^{3/2}={\left(1+\frac{0.75\left(\varphi /\phi_{m}\right)}{1-\frac{\varphi }{\varphi_{m}}}\right)}^{2}$	Model underpredicts severely
P14, Equation (38), new model, this study	−381.75%	$\eta_{r}{\left[\frac{2\eta_{r}+5\lambda }{2+5\lambda }\right]}^{3/2}={\left(\frac{1-\varphi }{1-\frac{\varphi }{\varphi_{m}}}\right)}^{2.5\varphi_{m}/\left(1-\varphi_{m}\right)}$	Model overpredicts extremely
FH, Equation (27), Faroughi and Huber [18]	${-1.30\times 10}^{8}\%$	${\eta_{r}=\left[\frac{\varphi_{m}-\varphi }{\varphi_{m}(1-\varphi )}\right]}^{-N\varphi_{m}/\left(1-\varphi_{m}\right)}$ where $N=\left(\frac{2+5\lambda }{2+2\lambda }\right)$	Model overpredicts extremely
P2, Equation (17), Pal [15]	${-3.9\times 10}^{24}\%$	$\eta_{r}{\left[\frac{2\eta_{r}+5\lambda }{2+5\lambda }\right]}^{3/2}=exp\left(\frac{2.5\varphi }{1-\varphi /\varphi_{m}}\right)$	Model overpredicts extremely

## Data Availability

The data presented in this study are available on request from the author.

## References

[1] Pal R. (2007). Rheology of Particulate Dispersions and Composites.

[2] Zhu Y., Gao H., Liu W., Zou L., McClements D.J. (2019). A review of the rheological properties of dilute and concentrated food emulsions. J. Texture Stud..

[3] Larson R.G. (1999). The Structure and Rheology of Complex Fluids.

[4] Borwankar R.P., Case S.E. (1997). Rheology of emulsions, foams and gels. Curr. Opin. Colloid Interface Sci..

[5] Alam M.M., Lemoto S., Aramaki K., Oshimura E. (2014). Self assembly and rheology of emulsions-mimicking food emulsion rheology. Food Struct..

[6] Serdaroglu M., Ozturk B., Kara A. (2015). An overview of food emulsions: Description, classification and recent potential applications. Turk. J. Agric.-Food Sci. Technol..

[7] McClements D.J. (2015). Food Emulsions-Principles, Practices, and Techniques.

[8] Rao M.A. (1999). Rheology of Fluid and Semisolid Foods.

[9] Taylor G.I. (1932). The viscosity of a fluid containing small drops of another liquid. Proc. R. Soc. Lond. A.

[10] Frankel N.A., Acrivos A. (1970). The constitutive equation for a dilute emulsion. J. Fluid Mech..

[11] Oldroyd J.G. (1953). The elastic and viscous properties of emulsions and suspensions. Proc. R. Soc. Lond. A.

[12] Yaron I., Gal-Or B. (1972). On viscous flow and effective viscosity of concentrated suspensions and emulsions. Rheol. Acta.

[13] Choi S.J., Schowalter W.R. (1975). Rheological properties of nondilute suspensions of deformable particles. Phys. Fluids.

[14] Pal R. (2000). Viscosity-concentration equation for emulsions of nearly spherical droplets. J. Colloid Interface Sci..

[15] Pal R. (2001). Novel viscosity equations for emulsions of two immiscible liquids. J. Rheol..

[16] Pal R. (2001). Single-parameter and two-parameter rheological equations of state for nondilute emulsions. Ind. Eng. Chem. Res..

[17] Mendoza C., Santamaria-Holek I. (2010). Rheology of concentrated emulsions of spherical droplets. Appl. Rheol..

[18] Faroughi S.A., Huber C. (2015). A generalized equation for rheology of emulsions and suspensions of deformable particles subjected to simple shear flow at low Reynolds number. Rheol. Acta.

[19] Brouwers H.J.H. (2010). Viscosity of a concentrated suspension of rigid monosized particles. Phys. Rev. E.

[20] Pal R. (2016). Modeling the viscosity of concentrated nanoemulsions and nanosuspensions. Fluids.

[21] Pal R. (2017). Viscosity-concentration relationships for nanodispersions based on glass transition point. Can. J. Chem. Eng..

[22] Pal R. (2020). New generalized viscosity model for non-colloidal suspensions and emulsions. Fluids.

[23] Thomas D.G. (1965). Transport characteristics of suspensions: VIII. A note on the viscosity of Newtonian suspensions of uniform spherical particles.. J. Colloid Sci..

[24] Graham A.L. (1981). On the viscosity of suspensions of solid spheres. Appl. Sci. Res..

[25] Pal R. (2015). A new model for the viscosity of asphaltene solutions. Can. J. Chem. Eng..

[26] Pal R. (1992). Rheological properties of emulsions of oil in aqueous non-Newtonian polymeric media. Chem. Eng. Commun..

[27] Pal R. (1992). Rheology of polymer-thickened emulsions. J. Rheol..

[28] Hsieh I.T.G. (2000). On-Line Viscosity Measurement of Emulsions. Master’s Thesis.

[29] Pal R. (1987). Emulsions: Pipeline Flow Behavior, Viscosity Equations, and Flow Measurement. Ph.D. Thesis.

[30] Pal R. (2000). Shear viscosity behavior of emulsions of two immiscible liquids. J. Colloid Interface Sci..

[31] Leviton A., Leighton A. (1936). Viscosity relationships in emulsions containing milk fat. J. Phys. Chem..

[32] Bains U. (2018). In-Situ Continuous Monitoring of Catastrophic Phase Inversion and Viscosity of Pickering Emulsions. Master’s Thesis.

[33] Buhidma A. (1995). Flow Measurement of Two-Phase Oil-in-Water Emulsions Using Wedge Meters and Segmental Orifice Meters. Master’s Thesis.

[34] Wolf B., Lam S., Kirkland M., Frith W.J. (2007). Shear-thickening of an emulsion stabilized with hydrophilic silica particles. J. Rheol..

[35] Pal A., Pal R. (2022). Rheology of emulsions thickened by starch nanoparticles. Nanomaterials.

[36] Bains U., Pal R. (2019). In-situ continuous monitoring of the viscosity of surfactant-stabilized and nanoparticles-stabilized Pickering emulsions. Appl. Sci..

[37] Kinra S., Pal R. (2023). Rheology of Pickering emulsions stabilized and thickened by cellulose nanocrystals over broad ranges of oil and nanocrystal concentrations. Colloids Interfaces.

[38] Van der Waarden M. (1954). Viscosity and electroviscous effect of emulsions. J. Colloid Sci..

[39] Tanner R.I. (2018). Review Article: Aspects of non-colloidal suspension rheology. Phys. Fluids.

[40] Pal R. (2023). Recent progress in the viscosity modeling of concentrated suspensions of unimodal hard spheres. ChemEngineering.

[41] Pal R. (1996). Effect of droplet size on the rheology of emulsions. AIChE J..

[42] Pal R. (2003). Viscous behavior of concentrated emulsions of two immiscible Newtonian fluids with interfacial tension. J. Colloid Interface Sci..

[43] Rust A.C., Manga M. (2002). Effects of bubble deformation on the viscosity of dilute suspensions. J. Non-Newton. Fluid Mech..

[44] Stein D.J., Spera F.J. (2002). Shear viscosity of rhyolite-vapor emulsions at magmatic temperatures by concentric cylinder rheometry. J. Volcanol. Geotherm. Res..

